# Means of Arresting Hæmorrhage from Leech Bites

**Published:** 1844-11-02

**Authors:** 


### MEANS OF ARRESTING HÆMORRHAGE FROM LEECH BITES.

The "Journal de Chirurgie" contains the details of an interesting case, narrated by Dr. Bordes, in which the twisted suture was successfully used to arrest hæmorrhage from leech bites. The operation is a trifling one, and, it appears, always successful, and consequently deserves to be better known. M. Bordes was called one evening to attend a young English lady, twenty-two years of age, who had had forty leeches applied to the abdomen at seven o'clock in the morning. Seven or eight of the leech bites were bleeding in the same manner as if veins had been opened with the lancet. She had lost all consciousness. Compression was impossible, and cauterization was not likely to succeed with so abundant a flow of blood. M. Bordes, recollecting the manner in which veterinary surgeons close the vein after bleeding horses, resolved to try the effect of the twisted suture. Pinching the skin at the orifice of the wound, he passed a small needle through it, and then tied a thread around. This slight operation was repeated for each orifice, and effectually arrested the bleeding. It was only the following day that the lady recovered her senses, and the convalescence lasted three months. M. Bordes has since frequently resorted to this plan and always with success.

M. Morand quoted in the "Journal de Medécine," has lately proposed another plan for arresting hæmorrhage from leech bites. He forms a small ball of a a mixture of olive oil and yellow wax, six parts of the first to one of the last, and, after wiping the blood from the wound, he rapidly applies it to the bleeding orifice. Pressing on it with his finger, he then spreads it around. If adhesion does not immediately take place, and the blood continues to flow, he adds a sufficient quantity of the oily mixture to form a cake, two-thirds of an inch in thickness, covering all the leech bites. The first time M. Morand tried this plan was on a child four years of age, attacked with severe pleurisy, who had had ten leeches applied to the chest. Several of the leech bites continued to bleed in spite of various remedies that had been tried. The oil and wax mixture at once arrested the hæmorrhage.—*Ibid.*

